# Supplementary material for: Are carbon emissions trading and green financial instruments synergistic? -Comprehensive quantitative research based on content analysis
Source: PLoS One. 2024 Mar 7;19(3):e0298601. doi: 10.1371/journal.pone.0298601 (PMC10919601; doi:10.1371/journal.pone.0298601)
Supplement: S1 Data — (ZIP) [file pone.0298601.s001.zip › Data/地级市Z_F工作报告.zip!/║╙▒▒/╠╞╔╜╩╨/└┤╫╘╣1⁄2╓┌║┼í╛┬φ┐╦╩2╛▌═°í┐_2014.docx]

一、2013年工作回顾

过去的一年，是本届政府的开局之年,也是唐山发展史上极不寻常的一年，经济发展遇到了多年来少有的困难和挑战。面对严峻形势和繁重任务，全市人民在省委、省政府和市委的坚强领导下，围绕建设沿海强市、美丽唐山的目标，坚定信心，顶住压力，攻坚克难，负重奋进，力促经济在困境中前行、在调整中发展，实现了稳中有进、稳中向好。全市地区生产总值完成6121.2亿元，增长8.3%;固定资产投资3575.9亿元，增长18.5%;公共财政预算收入318.4亿元，增长5.8%;社会消费品零售总额1724.6亿元，增长13.6%;城镇居民人均可支配收入26647元，农民人均纯收入11937元，增长9.4%和11.6%。在极其严峻的特殊形势下，我们始终坚信：没有走不出的低谷，只有越陷越深的沼泽。为了尽快突出重围，摆脱困境，闯出唐山发展的新天地，着力抓了以下工作：

一是着力推动产业转型升级。针对产业链条短、产品档次低、竞争力不强的实际，先后召开了冶金行业、服务业和金融业发展座谈会，对钢铁、焦化、建材等传统产业进行把脉会诊，分门别类制定发展规划和调整提升的路线图，下大力抻长产业链、提升价值链。全市重点项目完成投资中，耗钢产业投资占25.6%，消耗本地钢材占粗钢产量的14.5%，比上年增长了1.4倍。实施焦化产业链延伸项目16项,年可新增销售收入118.8亿元。完成工业技改投资1134.9亿元，增长24.8%。谋划实施战略性新兴产业项目141项，高新技术产业增加值增长20%。服务业投资占固定资产投资的49.8%，省级物流产业园区增至10家。新增市级农业龙头企业91家，农业产业化经营率达到67.5%。制定了培育市场主体的扶持政策，新增各类市场主体2.7万户，增长11.7%。

二是着力加速沿海经济发展。沿海地区生产总值、公共财政预算收入分别占全市的30%和32.2%，比上年分别提高1个和2.3个百分点。特别是举全市之力打造曹妃甸重要增长极，实施亿元以上项目170个，上汽绿色新能源汽车等15个项目竣工，中石化千万吨级炼油和华润二期项目前期工作取得突破性进展。曹妃甸综合保税区封关运营，唐山港货物吞吐量达到4.46亿吨，在全国港口排名上升到第7位。临港商务区宝骏金融街等30个服务业项目加快推进，唐山湾生态城基础设施和公共服务功能进一步完善，唐山工职院新校区建设取得阶段性成果，河北联合大学搬迁工作扎实推进。曹妃甸区完成固定资产投资734.9亿元，增长19.1%;实现公共财政预算收入48.7亿元，增长62.8%。

三是着力推进城乡一体化发展。全面提速2016年世园会筹办工作，完成世园会总体规划、详细规划及景区景观等专项规划编制，会址控制范围内的拆迁改造和配套建设全面展开。新建改造学院北路等14条城市道路，启动南湖水厂等一批市政设施建设，新火车站及东广场正式投入使用。基本完成了各县(市)区城乡总体规划编制工作，进一步完善了相关功能，基础设施投入达66.6亿元，县域经济达到全市经济总量的75%，城镇化率提高到54.4%。投资8.2亿元，以饮水安全、道路硬化、危房改造特别是农村旱厕无害化改造为重点，大力实施农村面貌改造提升行动，农村生产生活条件进一步改善。

四是着力深化改革开放。对高新区、路北区等8个县区的管辖范围进行调整，进一步理顺了管理体制，释放了发展活力;深入推进分税制财政体制改革，在全省率先取消全部财政收入考核，对有效化解历史遗留问题起到了积极的促进作用，为企业发展减轻了压力，调动了各县(市)区发展经济、增加地方财力的积极性;从完善区域金融体系入手，引进燕赵财险、西南证券等金融机构落户唐山，新获批村镇银行4家，新增上市公司11家。行政审批、科技创新、医药卫生等重点领域的改革取得了一定进展。广泛开展对接北京产业转移“招商月”系列活动，实际利用外资13.5亿美元，增长9.9%;进出口总额126.7亿美元，增长20.8%。

五是着力改善发展环境和生态环境。把环境建设作为加快发展的命门之穴，最大限度地汇聚正能量、增强区域竞争力。在发展环境上，从治理公路“三乱”入手，彻底取消上路罚款，交通环境得到明显改善;全市审批事项由238项调减到163项，成为全省审批事项最少的城市之一。在生态环境上，制定了大气污染综合治理65条措施，集中开展了五个行业污染治理、市区及周边重点污染企业搬迁改造等系列行动，先后关停重污染企业1085家，压减炼铁产能362万吨、炼钢产能620万吨，淘汰水泥产能226万吨、焦化产能315万吨，取缔燃煤锅炉336台，淘汰“黄标车”4.6万辆。通过集中攻坚，全市上下在大气污染防治上形成了高度共识，治理成果逐步显现，空气质量呈现好转态势。

六是着力保障和改善民生。在财税增收难度持续加大的情况下，市财政安排民生支出64.5亿元，占公共财政预算支出的73.2%。年初确定为群众办的十件实事全面完成。城镇新增就业10万人，登记失业率控制在4.02%。提高城乡低保标准、计划生育特殊家庭生活补助标准，启动实施了新农合大病保险。开工建设保障性安居工程1.6万套、竣工4.2万套。改建和增设农村幼儿园269所，完成中小学校舍安全工程。乡村卫生服务一体化覆盖率达到100%，新工人医院、新妇幼医院建设进展顺利。基层公共文化服务体系进一步健全，文化广场、新科技馆全面开工。信访、综治、维稳工作扎实有效，安全生产和食品药品安全监管工作不断加强。民族宗教、外事侨务、妇女儿童、审计、统计、体育、老龄、气象、档案、人防、防震减灾等各项工作都取得了新的成绩。

在推进经济社会发展的同时，大力加强政府自身建设，从严落实中央“八项规定”和省、市委相关要求，清理违规公务用车2038辆、超标办公用房7.5万平方米、“吃空饷”人员4010名，“三公”经费压减14.3%;认真执行市人大及其常委会各项决议，办理人大代表建议178件、政协委员提案494件，按时办复率均为100%。

各位代表，在经济形势非常严峻的情况下，取得这样的成绩来之不易。这是省委、省政府和市委正确领导的结果，是全市人民同心同德、拼搏奋进的结果。在此，我代表市政府，向全市人民，向人大代表、政协委员、各民主党派、工商联、无党派人士、人民团体和各界人士，向驻唐部队、武警官兵、公安政法干警，向关心支持唐山发展的老领导、老同志，向所有为唐山发展作出贡献的同志们、朋友们，表示崇高的敬意和衷心的感谢!

在肯定成绩的同时，我们也清醒地看到，尽管付出了很大的努力，但仍有地区生产总值、第三产业增加值、全部财政收入等部分指标完成得不够理想。经济发展中还存在不少矛盾和问题，主要表现在：结构性矛盾依然突出，转型升级任务异常繁重，特别是化解过剩产能压力巨大;部分企业生产经营困难，效益下滑态势尚未有效遏制;金融支撑不足，导致企业难以良性发展;大气污染防治任务艰巨，环境约束日益趋紧;政府职能和工作作风需要加快转变，一些部门和工作人员改革创新能力不强，服务意识、办事效率与企业和群众的期待还有一定差距。对此，我们一定高度重视，采取有效措施，认真加以解决。

二、2014年工作安排

今年是贯彻落实党的十八届三中全会精神、全面深化改革的开局之年，也是全面建成小康社会的关键一年。政府工作的总体思路是：深入贯彻落实党的十八届三中全会、省委八届六次全会和市委九届五次全会精神，坚持稳中求进工作总基调，把加速转型升级作为根本任务，把改革创新贯穿于经济社会发展的各个领域、每个环节，把新型城镇化作为发展的重大潜力，着力抓好项目建设，着力扩大开放招商，着力提速沿海开发建设，着力加强生态环境治理，着力保障和改善民生，加快全面建成小康社会步伐，奋力开创沿海强市、美丽唐山建设新局面。主要预期目标是：地区生产总值增长8%以上，全社会固定资产投资增长18.5%，公共财政预算收入增长10%以上，实际利用外资增长6%，社会消费品零售总额增长13%。单位生产总值能耗降低率、主要污染物削减量和城市环境空气质量改善率完成省达目标要求。城乡居民收入均增长8.5%，居民消费价格涨幅控制在3.5%左右。城镇登记失业率控制在4.5%以内，人口自然增长率控制在6.3‰以内。

实现上述目标，虽然有困难、有压力，但我们更有信心、更有决心。只要信心不滑坡，办法总比困难多。我们迎来了新的发展机遇。党的十八届三中全会拉开了全面深化改革的大幕，必将极大地激发我市经济社会发展的内生动力和活力;京津冀协同发展步伐加快，国家在重大项目布局上将曹妃甸列为重要增长极，为我们承接产业转移、吸纳先进生产要素提供了难得机遇。我们有干事创业的浓厚氛围。在应对复杂局面和严峻形势的实践中，各级领导班子和广大干部群众不畏艰难，敢于担当，奋力推动各项工作实现新突破，形成了千帆竞发、万马奔腾的生动局面。我们有弥足珍贵的唐山精神。尤其是“特别能战斗”的精神，集中彰显了唐山干部群众的精气神，引领全市上下在各个时期创造了一个又一个发展奇迹。以这样的精神抓改革、促发展，咬定青山不放松，不达目的不罢休，我们就一定能够战胜一切艰难险阻，再创唐山经济社会发展的新辉煌!

做好今年的政府工作，要正确处理好发展速度与转型升级的关系、环境治理与经济发展的关系、化解过剩产能与科学发展和社会稳定的关系，全力抓好“四个主题年”行动。一是深化改革攻坚年。全面推进“五位一体”各个领域的改革，坚决革除一切落后和阻碍发展的观念、做法和体制机制，为转型发展扫清障碍、奠定基础。二是结构调整提速年。真正把发展的重点放到提质增效升级上来，加快转方式、调结构步伐，延伸产业链，提升价值链，倒逼落后产能尽快退出唐山的生产领域，力促我市经济实现新的跨越。三是项目建设突破年。坚持一二三产并举、内资外资联动，加大项目谋划实施力度，形成大项目顶天立地、中小项目铺天盖地的生动格局。四是发展环境优化年。按照办事高效率、服务人性化的要求，注重从源头根治影响发展环境的突出问题，使唐山真正成为体制顺、机制活、审批快、效率高、成本低的投资热土和服务高地。

按照上述思路、目标和要求，重点抓好以下十个方面的工作：

(一)突出问题导向和活力激发，加快改革创新步伐。改革是时代的最强音，是发展的源动力。要把改革创新作为转型发展的开路先锋，发挥经济体制改革的引领作用，选准引爆点和突破口，着力破解发展难题，全面激发市场活力。一是加快国有企业改革。我市市本级国有资产总额1546亿元，其中，国有及国有控股企业总资产1441亿元。要制定出台国企改革方案，组建专门班子，多措并举，一鼓作气，确保6月底前完成改革任务。要鼓励引导国有资本、民营资本、央企、外企等交叉持股、融合发展，该转让的转让，该下放的下放，该重组的重组，努力实现股权多元化，彻底解决国有企业机制不活、管理僵化、竞争乏力、资产流失、经营困难等弊端和问题。二是激发民营企业创新发展活力。进一步清理规范现有政策，废除对民营经济的一切不合理规定。加快投融资体制改革，实行投资主体零限制、注册登记零收费。建立健全中小民营企业信用体系、融资担保机制，有效缓解民营企业融资难问题。加强对重点民营企业的帮扶，支持有条件的企业上市融资。三是推进行政管理体制改革。加大简政放权力度，能由市场调节完成的一律取消审批，能交给县级审批的一律下放到位，能备案的一律按备案执行。对应保留的审批事项，进一步优化审批流程，大力度削减审批要件，确保“窗口”一站办结。四是积极推进社会事业改革。鼓励引导社会资本进入教育、医疗、文化、健康、养老和公用事业等领域，逐步形成管办分离、多种所有制竞相发展的新格局。实施教育综合改革，深化医药卫生体制机制改革，探索实行政府购买公共服务新模式。五是着力破解要素瓶颈制约。支持符合条件的民间资本依法设立民营银行、金融租赁公司、担保公司、投资公司和消费金融公司，增强金融对地方经济的支撑带动作用。加快建立城乡统一、阳光透明的建设用地交易市场，探索推进农村集体经营性建设用地和宅基地上市交易、租赁、入股等。加强政府性债务管控，有效防范债务风险。

(二)突出项目建设和开放招商，加快培育新的经济增长点。项目决定成败，今天的项目就是明天的实力。今年，集中开展千个项目攻坚行动，其中新开工和在建项目800个，年度完成投资2000亿元。一是大中小项目齐抓。谋划实施一批引领产业方向、拉动税源增长、支撑一方发展的大项目、好项目，挺起唐山崛起的新脊梁。市级抓好唐钢高强汽车板、迁安正大精品管材等30个投资50亿元以上的重大产业项目;经济总量靠前的县(市)区，至少谋划实施10个投资10亿元以上的项目，其他县(市)区谋划实施5个以上。坚持抓大不放小，充分激发既有企业和民间资本的创业活力，让唐山的市场主体多起来、活起来、强起来。全年新增各类市场主体2.8万户。二是强力推进开放招商。牢固树立全域开放意识，大打开放牌，以开放促调整、促改革、促发展。尤其要用好国家放宽外商投资准入的新机遇，全方位加大招商引资力度，着力引进一批战略投资者。以北京产业加速转移为契机，主动加强对接合作，努力打造首都功能疏解的新空间、要素转移的新基地。年内，突出抓好200个投资5亿元以上的重点招商项目。项目靠招商，招商靠队伍。每个县(市)区招商局专职招商人员都要达到30人以上，并确保发挥应有作用。三是做精做强园区。按照小机构、大服务和集约型、集群化的思路，对现有园区和开发区科学定位，择优扶持，将其打造成重大项目的聚集地、延伸产业链条的主阵地和转型发展的新高地，全面释放园区发展的新能量。市级重点抓好曹妃甸工业区、高新区、海港开发区、开平现代装备制造工业区和迁安西部工业区，确保固定资产投资等主要经济指标增长30%以上。年内主营业务收入超100亿元的园区要达到22个，超500亿元的7个，超1000亿元的2个。

(三)突出有中生新和无中生有，加快推进工业转型升级。经济困难时期，更是转型升级的最佳时机。要切实把工业转型升级作为经济工作的核心任务，按照“改造旧的、发展新的、培育好的”要求，坚持“有中生新、无中生有”两手抓，尽快做强“长板”、补齐“短板”。一是加快延伸产业链条。没有夕阳的产业，只有夕阳的产品。针对我市传统工业“有产业缺市场、有产品缺商品”的现状，制定落实推动精深加工和商品开发的扶持政策，加快产品由中端向末端、由末端向商品的迈进，让更多的“唐山制造”、“唐山创造”走进千家万户。年内，每个县(市)区都要谋划1-2个商品制造园区，全市新发展耗钢企业500家以上、商品制造企业200家以上，消耗本地钢材达到粗钢产量的20%左右。全年安排技改专项资金1亿元，实施重点技改项目500项。二是坚定有序化解过剩产能。旧的不去，新的不来。年内计划压减炼铁产能413万吨、炼钢产能1082万吨，淘汰水泥产能485万吨。这是一项硬指标、硬任务，必须不折不扣地完成。同时，正确处理好“减”与“增”、“退”与“进”的关系，通过腾笼换鸟和创新驱动，努力在调整中形成新的产业和竞争力。今后的唐山，“两高”项目一个不上，过剩产能一吨不增，低效益投资一概拒之门外!三是大力发展战略性新兴产业。抢抓新一轮科技革命和产业变革孕育兴起的机遇，进一步壮大新能源、节能环保、生物医药、电子信息等产业规模，促其迈向高端化、规模化和集群化。年内，确保轨道客车扩能、汉能薄膜太阳能发电等20个重大项目取得实质性进展，战略性新兴产业增加值增长20%以上。四是做大做强行业龙头。针对我市企业“缺大少强、群山无峰”的现状，大力推动企业二次创业，组织实施“百企培育”计划，加快打造一批企业航母和产业巨人。到年底，年营业收入超10亿元的企业达到200家，超50亿元的50家，超100亿元的30家，超500亿元的6家以上，支持庞大汽贸、丰润动车城、冀东集团、首钢唐山产区、唐钢集团等企业和集群早日迈进营业收入“千亿俱乐部”。狠抓企业上市工作，年内新增上市公司15家以上，每个县(市)区要新发展1至2家。按照新的品牌评价国家标准，引导企业争创中国星级品牌，新培育中国驰名商标4件、省著名商标40件。五是提升科技支撑能力。依靠科技创新为企业强筋壮骨，不断提升企业的生命力。今年，市级安排科技贴息资金500万元，新建市级以上大型企业研发中心30个，培育科技型中小企业400家，实施重点科技成果转化项目50项。力争经过三年努力，超过70%的规模以上企业建立研发机构。适应转型升级需要，围绕经济结构调整和新兴产业发展，加大人才培养引进力度，年内新增特邀院士10名，引进高层次、高技能紧缺人才1万名。

(四)突出总量扩张和功能拓展，加快发展服务业。我市服务业规模较小、比重偏低，发展相对滞后。今年，要多措并举，从规划、政策、要素配置等方面入手，强力推动服务业大发展、快发展。年内服务业增加值确保增长10%。一方面，推动生活性服务业提档升级。坚持高端引领、中端支撑、多元配套，以满足群众多层次、多样化的消费需求为导向，大力发展城市经济，加快改造提升商业购物、旅游休闲、健康养老、餐饮娱乐等产业。突出抓好总投资295.5亿元的华润万象城、勒泰商城、凤城国贸等10个重点商业综合体项目，引进一批世界名品名店，打造一批现代大型卖场，建设一批特色商贸街区，不断提升城市的繁华度。鼓励发展我市电子商务、网络购物、邮政快递等新兴服务业态，让群众足不出户就能尽享现代购物的便利。加快唐山湾国际旅游岛、曹妃甸湿地文化旅游度假区、清东陵保护利用等10大旅游项目建设，力争新增5A级景区1家、4A级景区3家。完善落实扶持政策，统筹推进教育、文化、医疗、养老等产业发展，满足群众精神文化和健康需求。另一方面，推动生产性服务业提速壮大。做大做强现代物流、金融保险、科技研发、工业设计等产业，促进制造业与服务业的快速融合发展。着力抓好总投资229亿元的天津物产综合服务中心等20个重点物流项目，加快建设一批仓储物流基地。大力发展第三方物流，促进企业内部物流社会化，支持民营企业广泛进入物流领域，全面提升我市物流产业发展的水平和档次。深入挖掘铁路内在潜力，尽快整合优化既有铁路资源，规划启动市区和曹妃甸两个编组站以及岔河、贾庵子、古冶、迁安等枢纽站建设，形成布局合理、功能完备、快捷高效的铁路运输网络，谋划实施一批重点物流园区，有效降低物流成本，缓解公路运输压力。培育壮大金融市场主体，抓好路北区金融中心建设，大力发展证券、担保、融资租赁等多种金融业态。财政拿出专项补贴资金，支持各类金融主体加快发展。

(五)突出战略摆位和全面小康，加快让农业强起来、农村美起来、农民富起来。唐山是农业大市，要基本实现2016年全面建成小康社会的目标，基础在农业，难点在农村，关键在农民。坚持工业反哺农业、城市支持农村和多予少取放活的方针，全面落实强农惠农政策，让广大农民平等参与现代化进程、共同分享现代化成果。一是着力培育壮大农业龙头。以标准化、规模化、集约化生产为方向，推动龙头企业上规模、上档次，大力支持现代农业产业园区建设。今年，市级重点抓好100家龙头企业晋档升级，每个县(市)区至少新发展5家以上，全市农业产业化经营率达到68.5%。建设完善标准化规模养殖示范场(小区)320个，新建国家级水产健康养殖示范场5个、现代农业示范园区20个。农业重点县区至少新增种养大户40个、家庭农场40个、农民合作联合社2个。二是着力实施农村面貌改造提升行动。在巩固去年工作的基础上，再选定380个重点村，大力实施改水、改厨、改厕、绿化等工作，新建大中型沼气工程91处，新增节水灌溉面积20万亩。集中力量抓好山水林田湖生态修复，全面提升农村人居环境，努力打造保持田园风光、融入现代生活的美丽乡村。三是着力保障农产品质量安全。加快发展绿色农业、有机农业，健全农业标准化生产体系，完善覆盖从田间到餐桌全过程的监管制度。年内标准化生产种植面积达到700万亩以上，农产品抽检合格率达到98%以上。四是着力拓宽农民增收渠道。在提高农业综合效益的同时，因地制宜发展中小企业和家庭手工业，不断增加农民的工资性收入。深入开展百万农民大培训和农村劳动力阳光培训工程，全年培训农民150万人次，为农民进城、就业创业开辟新的渠道。同时，以土地制度改革为核心，稳妥推进农村综合改革，为农业农村发展注入生机和活力。

(六)突出潜能释放和产业聚集，加快沿海一线在全市率先崛起。目前，我市沿海开发建设呈现出加速崛起的良好态势，但总体看，黄金宝地、深水大港和全球知名的优势还没有充分释放出来。今年，要进一步提振精神、鼓足干劲，举全市之力，集各方之智，聚焦沿海，会战沿海，强力推动以曹妃甸为龙头的沿海开发建设实现大跨越、大提升，使之尽快成为全市乃至全省经济发展的领跑者。年内，沿海地区重点项目数量、固定资产投资要达到全市的45%以上;曹妃甸区固定资产投资增长22%，公共财政预算收入确保增长30%、力争达到50%。一是全面提速项目建设。以攻坚之势开展项目建设大会战，加快形成庞大的产业集群，撑起沿海发展的一片天。重点抓好500个亿元以上项目，争取中石化千万吨级炼油、华润二期开工建设，强力推进首钢二期、大型海水淡化等一批重大产业项目取得实质性进展。同时，狠抓中小项目建设，全面增强沿海经济的发展活力。二是加快打造综合贸易大港。在抓好各类泊位建设和运营的基础上，尽快启动曹妃甸五号、六号港池和京唐港区集装箱、丰南港区通用码头建设。全面提升港口功能，年内建成矿石、煤炭和木材三大交易中心，充分释放综合保税区的优势，推进大宗过境商品就地深加工和物流园区建设，力促综合贸易大港建设实现新突破。唐山港货物吞吐量达到4.7亿吨以上、集装箱突破100万标箱。延伸和拓展港口腹地范围，打造华北、西北地区乃至蒙古国的出海口。三是着力抓好港城建设。围绕把唐山湾生态城打造成唐山南部次中心城市，把临港商务区建成曹妃甸工业区的生活保障区，加快完善基础设施和服务功能，确保唐山工职院新校区今年秋季开学，全面提速河北联合大学搬迁进程，确保2015年秋季招生，为曹妃甸聚人气、增活力。围绕增强区域发展的承载能力，加快遵曹公路、滨海公路建设进度，唐曹公路竣工通车，迁曹高速曹妃甸段力争3月底前开工，唐曹铁路、水曹铁路上半年开工，张唐铁路完成线下工程。

(七)突出综合施策和源头治理，加快提高生态环境质量和水平。人的生命不可复制，人类生存既要追求生命的长度，更要追求生存的质量，享有优质的生态产品，拥有良好的生态环境。要以坚定的决心，一鼓作气，一抓到底，坚决打好大气污染防治攻坚战。一是突出抓治污。坚持以市区为重、点面结合，深入开展钢铁等五个行业大气污染治理攻坚行动，制定出台能耗和排放标准，2015年全面完成高标准脱硫、脱硝和除尘改造。市区及周边重点污染企业搬迁改造必须坚定不移加以推进，确保年内取得突破性进展，其中，唐钢炼焦制气3月中旬实施停产搬迁。抓好第三批严重污染企业及落后装备专项治理，巩固好一、二批治理成果。全面关闭市中心城区企业自备水井，中水利用率达到50%以上。年底前市区高耗水企业全部使用中水，尽快实现地下水零开采。二是重点抓减煤。深入实施天然气进城、下乡、上高速和“县县通”工程，年内各县(市)区城区居民集中气化率要达到90%以上，规模以上工业企业燃气消费总量增长30%以上。全面放开天然气批发、零售市场，新建一批加气站，有效解决“加气难”的问题。狠抓市区燃煤锅炉淘汰工作。到年底，全市压减燃煤量430万吨。三是着力抓控车。加大环保公交车投放力度，确保年内全部更新到位。今后，新增公交车、出租车一律使用清洁燃料。加快“黄标车”淘汰步伐，强力推进大客车、货车“油改气”工程，力争三年内基本完成货运汽车气化改造。着手研究探索抑制小汽车过快增长的措施和办法。四是广泛抓增绿。按照“高大密厚”的标准，动员全社会力量大搞造林绿化，让绿树森林成为吸收大气污染物的天然“净化器”。重点抓好城市周边、企业、道路沿线和村庄绿化，对国道、高速公路、铁路沿线等区域破坏严重的山体，强化生态修复治理，尽快披上生态绿衣。年内，新增造林面积32万亩。五是从严抓管理。落实企业环保主体责任，实行五个行业“一企一证一卡”排污总量控制模式，全面启动惩罚性和差别价格措施。重点工业区和企业配齐空气质量监测装置，实行24小时在线监测。发挥好公安环境安全保卫支队的作用，严厉打击偷排偷放等违法行为。大气污染是在长期发展中积累形成的，彻底根治需要大量卓有成效的工作。只要我们上下一条心、拧成一股劲，一步一个脚印地抓下去，就一定能够早日换回人民期盼的碧水蓝天!

(八)突出功能完善和品位提升，加快打造与世园会相适应的城市新形象。举办一届有特色、高水平的世界园艺盛会，对于加快城市现代化和国际化步伐，提升唐山在全国乃至世界的影响力、竞争力、知名度和美誉度具有重大意义。今年，要以筹办2016年世园会为主线，集中力量打好城市建设攻坚战，加快建设宜居、宜业、宜商、宜学的现代化城市。一是推动筹办工作大提速。全面启动世园会场馆、展馆、展园建设，年内主体工程基本完工，明年全部竣工。加快推进会址及周边区域市政基础设施建设，力争年内完工。持续开展“绿、美、亮、净”综合整治，抓好城市主干道、出入口、公园、广场和重要节点的公共景观建设和绿化提升，做到三季有花、四季常绿、全年有景。特别要高标准规划改造迎宾大道景观，将其打造成富有现代气息、展示唐山形象的靓丽风景线。二是推动城市功能大完善。全面加强教育、医疗、文化、商业等公共服务设施建设，新建改造南新西道、站前路等一批城市道路。加快西郊电厂配套热网等市政建设，完成新火车站西广场主体工程。加大协调跑办力度，力争京唐高铁早日开工建设，尽快启动遵化至唐山南站城际铁路建设，争取年内客运投入运营。高标准规划建设一批宾馆酒店、餐饮娱乐、旅游休闲等服务设施，提高接待服务能力。三是推动城市管理水平大提升。探索推行“数字城管”新模式，实现全天候、全方位、全覆盖管理。坚持重心下移，推进物业管理权限下放，鼓励社会和公众参与城市管理。狠抓市容市貌管理，集中整治临街破门开店、破坏小区绿地、城乡结合部脏乱差等群众反映强烈的突出问题，3月底前完成严重超限超载车辆整治任务，6月底前彻底取缔市中心区客运三轮车，加快推进市区停车产业化发展，打造环境整洁、秩序井然、管理科学的城市新形象。

(九)突出规划引领和城乡统筹，加快新型城镇化进程。城镇化是现代化的必由之路。坚持因势利导、以人为本，构建特色鲜明、布局合理、功能完善的现代城镇体系。一是优化城镇空间布局。抓好新一轮城市总体规划修编，加快构筑以市中心区和唐山湾生态城为“双核”、以中小城市为“组团”的城镇化发展新格局。在确定的城市边界内，市中心区坚持南拓、东联并举，打造以南湖为核心的城市中心;唐山湾生态城要加快完善各项基础设施，加速人口聚集，尽快形成应有的辐射和带动效应。二是有序推进农业转移人口市民化。按照因地制宜、分步推进，存量优先、带动增量的原则，全面放开市区、县城和建制镇的落户限制，逐步把进城落户的农民纳入城镇社会保障体系，努力实现义务教育、就业服务、社会保障、基本医疗、保障性住房等全覆盖到城镇常住人口，实现“同城同权利、同城同待遇”。年内转移农村人口9万人，城镇化率提高到55.6%。三是加快完善城镇功能要素。按照市中心区300万、大县城30万、小县城15万、中心镇3至5万的人口规模，科学规划教育、医疗、文化、商业等公共服务设施，适度超前的规划实施交通、供水、供电、供气、供热、污水处理、垃圾处理等基础设施，年内启动一批建设项目，不断提升城镇的承载能力。四是大力推动县域经济发展。坚持分类指导，统筹资源要素，有选择、有侧重地加大支持力度，全面提升县域经济的实力、活力和竞争力。每个县至少新培育3个特色产业，打造一批产业集群,助推县域经济跨越发展。

(十)突出民生改善和社会建设，加快提升人民群众的生活质量和幸福指数。悠悠万事，民生为大。今年，市财政安排民生和社会事业资金73.1亿元，占公共财政预算支出的77.1%，重点围绕就业、安居、养老、健康、公共服务、社会保障等方面，实施一批民生项目，切实解决好群众关心关注的热点难点问题。就业社保方面：统筹推进各类群体就业，年内城镇新增就业9万人以上;全力推进社保扩面提标，完善医保市级统筹信息系统，加强社会养老服务体系建设，建立贫困重度残疾人生活补贴和贫困残疾儿童抢救性康复补贴制度。教育方面：加快推进各类教育均衡发展，实施名校、名师、名校长工程，抓好新开滦一中等重点项目建设，优化整合高等教育资源，科学布局初中、高中教育，努力打造教育强市。医疗卫生方面：深入开展“树名医、建名科、创名院”活动，推行全市医疗机构“一卡通”，提高新农合筹资标准，年内新工人医院、新妇幼医院正式投入使用。文化方面：完善基层公共文化设施，新科技馆和文化广场主体完工;培育壮大文化龙头企业，抓好唐山陶瓷文化创意中心等20个重点文化项目建设;鼓励各类资本进入文化领域、投资文化产业;深化文化单位体制机制改革，增强内在发展活力。体育方面：启动新奥体中心建设，城区新建3条健身步道、安装150套健身路径，所有行政村配齐体育健身设施。做好人口计生、民族宗教、外事侨务、广播电视、妇女儿童、老区建设、防震减灾、气象、档案等工作。深入开展群众性精神文明创建活动。加强国防动员和民兵预备役建设。

各位代表，加快发展任重道远，改善民生永无止境。今年，我们将继续为群众办好十件实事。一是着力实施大气污染防治工程，完成钢铁等五个行业128家企业的659个治理项目，新建加气站80座、年内建成60座，淘汰“黄标车”2.4万辆，购置环保公交车565辆，推广秸秆成型燃料炉具2万户并配套压块设备，切实减少农村空气污染。二是实施水源地保护和水质提升工程，让唐山市民喝上放心水、优质水;继续抓好农村饮水安全工程，解决400个村、35万农村人口的饮水安全问题。三是年底前开通铁路唐山站至北京站的客运专线，实现1小时直达进京。四是投资53.7亿元，启动城市二环路、唐古路和唐古快速路建设，解决客货车混行、交通拥堵、秩序混乱的问题;投资1.6亿元，新建改造农村公路400公里，以方便农村群众出行。五是财政投入资金，市中心区年内竣工保障性住房1000套、5.5万平方米，解决中低收入家庭住房问题。六是投资1.2亿元，启动建设唐山就业社保大厦，完善就业市场体系;建成40个标准化乡镇(街道)劳动就业社会保障服务所，为群众提供集中便捷的就业、医保、社保等公共服务。七是提高城乡低保标准，城市由每人每月450元提高到470元，农村由每人每年2900元提高到3150元。八是投资3.3亿元，改造农村旱厕37万座，提升农民生活质量。九是改建、增设农村幼儿园120所，提高农村学前教育水平。十是投资2.4亿元，建设1个放心食品“中央大厨房”、10个物流分拨中心、20个配送中心、500个配送门店，为群众解决“最后一公里”配送难的问题。在为民办实事上，我们将一诺千金，说到做到，把每件实事办成群众满意工程，努力让全市人民过上更加幸福美好的生活!

三、全面提升政府治理能力和服务水平

打铁还需自身硬。经济社会加速转型，政府治理方式必须加快转变。我们要以开展党的群众路线教育实践活动为动力，积极推进政府治理能力现代化，努力打造法治政府和服务型政府。

更加注重转变政府职能。要合理界定政府与市场、企业和社会的关系，将主要精力集中到加强发展战略、规划、政策、标准的制定实施和市场监管、公共服务、社会管理、环境保护上来，把不该管、管不了、管不好的权力，还权于企业、回归到市场、交还给社会、下放到基层，充分激发各个方面的活力。

更加注重维护公平正义。只有公平得以实现、正义得到伸张，百姓才会气顺，社会才能和谐。要围绕促进分配公平、就业公平、教育公平、医疗公平和司法公正，逐步缩小收入差距，不断增强公共产品和公共服务的供给能力，引导群众依法表达利益诉求，使全体人民在经济社会发展中更多更好地平等参与、平等竞争、平等发展、平等享有。全体政府工作人员要以实际行动维护公平正义，大力弘扬新风正气，坚决抵制歪风邪气，让公平正义的光辉普照唐山大地，让老百姓生活得不压抑、有尊严、更幸福。

更加注重创造良好发展环境。要痛下决心，像治理公路“三乱”那样，出重拳，用实招，力促发展环境大改善、效能大提速。今年要彻底解决行政审批环节多、效率低和“体外循环”的问题，集中根治“吃拿卡要”和“门难进、脸难看、事难办”等问题，从严惩处破坏发展环境的行为，确保唐山发展环境实现根本好转。要树立“企业创造财富、政府创造环境”的理念，多服务、少干预，多帮忙、少添乱，多设路标、少设路障，让各类市场主体放开手脚、竞相发展。扎实推进政府机关标准化建设，实现工作提速、服务提质。加强和创新社会治理，强化安全生产和食品药品安全监管，深入推进平安唐山建设，严厉打击各类违法犯罪行为，保障人民群众吃得放心、用得安全、住得舒适、睡得踏实。

更加注重依法行政。大力推进法治政府建设，营造政府依法行政、企业依法经营、市民依法行为、社会依法运转的良好氛围。严格执行重大行政决策程序规定，确保决策科学化、民主化。自觉接受人大、政协以及各界群众和新闻媒体的监督，认真贯彻执行市人大及其常委会的决议决定，切实办理好人大议案、代表建议和政协委员提案。

更加注重从严治政。扎实开展党的群众路线教育实践活动，集中解决“四风”问题，以良好的作风服务群众、造福百姓。健全和完善抓落实的机制，确保各项工作件件有着落、事事有结果。全面推进政务公开，强化对土地出让、国有资产产权交易、建设工程招投标、政府采购等重点领域的监管，发挥审计、监察的职能作用，让监督的阳光照亮权力运行的每一个环节。厉行勤俭节约，严格“三公”经费支出，在各级政府自觉过“紧日子”中，让群众过上“好日子”。

各位代表，奋斗成就梦想，实干创造辉煌。让我们紧密团结在以习近平同志为总书记的党中央周围，在省委、省政府和市委的坚强领导下，紧紧依靠全市人民，攻坚克难，开拓奋进，同心同德，大干快上，为加快建设具有实力、活力、魅力的沿海强市、美丽唐山而努力奋斗!
